# The Role of K_v_7 Channels in Neural Plasticity and Behavior

**DOI:** 10.3389/fphys.2020.568667

**Published:** 2020-09-18

**Authors:** Brian C. Baculis, Jiaren Zhang, Hee Jung Chung

**Affiliations:** ^1^Neuroscience Program, University of Illinois at Urbana-Champaign, Urbana, IL, United States; ^2^Department of Molecular Integrative Physiology, University of Illinois at Urbana-Champaign, Urbana, IL, United States

**Keywords:** KCNQ channel, K_v_7, intrinsic excitability, synaptic transmission, neural plasticity, learning, memory, behavior

## Abstract

Activity-dependent persistent changes in neuronal intrinsic excitability and synaptic strength are widely thought to underlie learning and memory. Voltage-gated KCNQ/K_v_7 potassium channels have been of great interest as the potential targets for memory disorders due to the beneficial effects of their antagonists in cognition. Importantly, *de novo* dominant mutations in their neuronal subunits *KCNQ2*/K_v_7.2 and *KCNQ3*/K_v_7.3 are associated with epilepsy and neurodevelopmental disorder characterized by developmental delay and intellectual disability. The role of K_v_7 channels in neuronal excitability and epilepsy has been extensively studied. However, their functional significance in neural plasticity, learning, and memory remains largely unknown. Here, we review recent studies that support the emerging roles of K_v_7 channels in intrinsic and synaptic plasticity, and their contributions to cognition and behavior.

## Introduction

Voltage-gate channel potassium (K^+^) subfamily Q member 1–5 (*KCNQ* 1–5) encodes K_v_7.1–K_v_7.5 channels (Gutman et al., 2005) that are critical regulators of excitability in neurons, muscles, and sensory cells (Soldovieri et al., 2011). All K_v_7 subunits have six transmembrane segments (S1–S6; Robbins, 2001). The S1–S4 comprise a voltage-sensing domain with the S4 being the main voltage-sensor (Robbins, 2001). The pore domain consists of the S5-S6 flanking the pore loop important for K^+^ ion selectivity (Sun and MacKinnon, 2017). The gate is formed by the intersection of four S6 segments (Cui, 2016; Sun and MacKinnon, 2017). Upon depolarization, the electric field on the basic residues of S4 promotes its translational rotation and outward displacement, which leads to the opening of the gate (Cui, 2016; Sun and MacKinnon, 2017). All K_v_7 channels require phosphatidylinositol-4,5-bisphosphate (PIP_2_) in the plasma membrane for channel opening (Zhang et al., 2003; Suh and Hille, 2008; Zaydman and Cui, 2014), and PIP_2_ is proposed to couple the voltage-sensing domain to the pore domain in K_v_7.1 (Zaydman et al., 2013; Sun and MacKinnon, 2020). Each K_v_7 subunit also has a short intracellular N-terminal domain and a long intracellular C-terminal tail that harbors four helices (helices A–D; Haitin and Attali, 2008). Helices A and B bind to calmodulin (CaM; Strulovich et al., 2016; Sun and MacKinnon, 2017), whereas helices C–D mediate subunit assembly (Haitin and Attali, 2008).

In neurons, K_v_7 channels open at subthreshold potentials around −60 mV and produce slowly-activating and non-inactivating outward K^+^ currents that potently suppress repetitive and burst firing of action potentials (APs; Brown and Passmore, 2009). Their functional significance in inhibiting neuronal excitability is underscored by the fact that mutations in their subunits cause epilepsy (Nappi et al., 2020), whereas K_v_7 agonist retigabine inhibits seizures in rodents and humans (Miceli et al., 2008). Importantly, emerging new evidence suggests that K_v_7 channels may contribute to activity-dependent persistent changes in neuronal intrinsic excitability and synaptic strength that are widely thought to underlie learning and memory. This review will summarize the function of K_v_7 channels in the hippocampus and discuss recent studies that investigate their contributions to hippocampal plasticity, cognition, and behavior.

## Brain Distribution of K_v_7 Subunits and Their Channelopathies

K_v_7.2, K_v_7.3, and K_v_7.5 are the major neuronal K_v_7 subunits (Table 1). K_v_7.2 and K_v_7.3 show strong overlapping expression in the cerebral cortex, hippocampal formation, amygdala, basal ganglia, and hypothalamus (Wang et al., 1998; Cooper et al., 2001; Klinger et al., 2011). K_v_7.5 is highly expressed in the brain stem and to less extent in the cerebral cortex, hippocampus, occipital, frontal, and temporal lobes (Lerche et al., 2000; Schroeder et al., 2000; Tzingounis et al., 2010; Fidzinski et al., 2015). While K_v_7.1 and K_v_7.4 are mainly found in the heart and cochlear hair cells, respectively (Wang et al., 1996; Kubisch et al., 1999), they are also detected at low level in multiple regions of the brain (Casimiro et al., 2001; Hansen et al., 2006; Goldman et al., 2009; Su et al., 2019; Table 1).

**Table 1:** Distribution of K_v_7 subunit in the brain and the diseases associated with its pathogenic variants.

Gene	Protein	Primary location	Distribution in the brain		Pathogenic variants	
			Regions	Reference	Associated diseases	Reference
*KCNQ1*	K_v_7.1	Heart	CTX, HPF, MB, CB, BS	Casimiro et al., 2001; Goldman et al., 2009. AIBS, THPA	Long QT syndrome 1, JLNS, familial atrial fibrillation epilepsy, SUDEP, ASD, developmental disorder	ClinVar, LOVD, *denovo*-db
*KCNQ2*	K_v_7.2	Nervous system	CTX, HPF, A, HY, TH, OA, MD, SN, P, MY, CB	Wang et al., 1998; Cooper et al., 2001; Devaux et al., 2004; Klinger et al., 2011; D’Este et al., 2016; Galvin et al., 2020. AIBS, THPA	BFNE, EE, ASD, intellectual disability, developmental disorder, sporadic infantile spasm syndrome	ClinVar, RIKEE, *denovo*-db
*KCNQ3*	K_v_7.3	Nervous system	CTX, HPF, A, HY, TH, OA, MD, SN, P, MY, CB	Wang et al., 1998; Devaux et al., 2004; Klinger et al., 2011; Galvin et al., 2020. AIBS, THPA	BFNE, EE, ASD, intellectual disability, developmental disorder	ClinVar, RIKEE, *denovo*-db
*KCNQ4*	K_v_7.4	Inner ear	BS, OA, MD, RN, NA, MY, VTA, P	Hansen et al., 2006; Su et al., 2019. AIBS, THPA	DFNA2, ASD	ClinVar, *denovo*-db
*KCNQ5*	K_v_7.5	Nervous system	CTX, HPF, BS, CB	Lerche et al., 2000; Schroeder et al., 2000; Tzingounis et al., 2010; Fidzinski et al., 2015; Galvin et al., 2020. AIBS, THPA	EE, ASD, intellectual disability, schizophrenia	ClinVar, RIKEE, *denovo*-db

Brain regions: CTX, cortex; OA, olfactory areas; HPF, hippocampal formation; A, amygdala; NA, nucleus accumbens; BS, brain stem; TH, thalamus; HY, hypothalamus; MB, midbrain; RN, raphe nuclei; SN, substantia nigra; VTA, ventral tegmental area; PAL, pallidum; HB, hindbrain; CB, cerebellum; P, pons; MY, medulla. K_v_7 channelopathies: JLNS, Jervell and Lange-Nielsen syndrome; SUDEP, sudden unexpected death in epilepsy; BFNE, benign familial neonatal epilepsy; EE, epileptic encephalopathy; ASD, autism spectrum disorder; DFNA2, nonsyndromic sensorineural deafness type 2. Database website: Allen Institute for Brain Science (AIBS, https://alleninstitute.org/what-we-do/brain-science/), The Human Protein Atlas (THPA, https://www.proteinatlas.org/), ClinVar (https://www.ncbi.nlm.nih.gov/clinvar/), Leiden Open Variation Database (LOVD, https://research.cchmc.org/LOVD2/home.php), *denovo*-db (http://denovo-db.gs.washington.edu/denovo-db/index.jsp), and Rational Intervention for KCNQ2/3 Epileptic Encephalopathy (RIKEE, https://www.rikee.org/).

Importantly, >300 dominant mutations in *KCNQ2* and *KCNQ3* cause epilepsy including benign familial neonatal epilepsy (BFNE) and epileptic encephalopathy (EE; Rikee and ClinVar database). *KCNQ2* is the second most frequently mutated gene in neurodevelopmental disorder (Traynelis et al., 2017; Coe et al., 2019) characterized by cognitive and behavioral deficits (Mullin et al., 2013). A few mutations in *KCNQ1*, *KCNQ4*, and *KCNQ5* have been associated with epilepsy, autism, schizophrenia, and developmental disorder (Table 1). Haploinsufficiency in K_v_7 function seems to underlie BFNE variants that cause the transient appearance of neonatal seizures (Soldovieri et al., 2011). EE patients display severe and often drug-resistant neonatal seizures and psychomotor retardation (Weckhuysen et al., 2012), and *de novo* EE mutations in *KCNQ2* and *KCNQ3* induce multiple defects in current and surface expression of K_v_7 channels (Weckhuysen et al., 2012, 2013; Milh et al., 2013; Miceli et al., 2015; Kim et al., 2018; Zhang et al., 2020).

## General Properties and Regulation of K_v_7 Currents

K_v_7.1 assembles with auxiliary β subunit KCNE1 to produce the slow delayed rectifier K^+^ current (I_Ks_) important for the repolarization of cardiac APs (Barhanin et al., 1996). Importantly, coassembly with KCNE1 slows the activation kinetics of K_v_7.1 channel, potentiates its current amplitude, and eliminates its voltage-dependent inactivation (Barhanin et al., 1996; Sanguinetti et al., 1996; Tristani-Firouzi and Sanguinetti, 1998). Homomeric K_v_7.2 channels activate at −60 mV and produce slow-activating and non-inactivating currents (Biervert et al., 1998). In comparison, currents through K_v_7.3 channels are negligible due to an Ala residue in the pore domain (Wang et al., 1998; Gomez-Posada et al., 2010). K_v_7.5 activates at −60 mV with slower kinetics than K_v_7.2 and K_v_7.3 (Schroeder et al., 2000; Gamper et al., 2003). K_v_7.4 activates at −40 mV with slower activation kinetics than other K_v_7 channels (Kubisch et al., 1999).

Neuronal K_v_7 channels are mostly heterotetrameric channels composed of K_v_7.2 and K_v_7.3, and to a lesser extent K_v_7.3 and K_v_7.5 (Wang et al., 1998; Shah et al., 2002; Table 1). Compared to homomeric channels, significantly larger currents are generated by K_v_7.2/K_v_7.3 channels (Schroeder et al., 1998; Wang et al., 1998; Schwake et al., 2000) and K_v_7.3/K_v_7.5 channels (Schroeder et al., 2000; Gilling et al., 2013). K_v_7.2/K_v_7.3 channels produce M-current (*I*_M_; Wang et al., 1998), which potently suppresses neuronal hyperexcitability (Wang et al., 1998; Yue and Yaari, 2004). *I*_M_ is inhibited by muscarinic acetylcholine receptor activation (Selyanko et al., 2000) and the depletion of PIP_2_ (Suh and Hille, 2002; Zhang et al., 2003). K_v_7 channels are also inhibited by other G-protein coupled receptors, including substance P, bradykinin, serotonin, angiotensin, luteinizing hormone-releasing hormone, opioid, and metabotropic glutamate receptors (Marrion, 1997). General properties and diverse regulation of K_v_7 channels are described in detail in a previous review (Soldovieri et al., 2011).

## Role of K_v_7 Channels in Intrinsic Excitability and Plasticity in the Hippocampus

**Figure 1:**
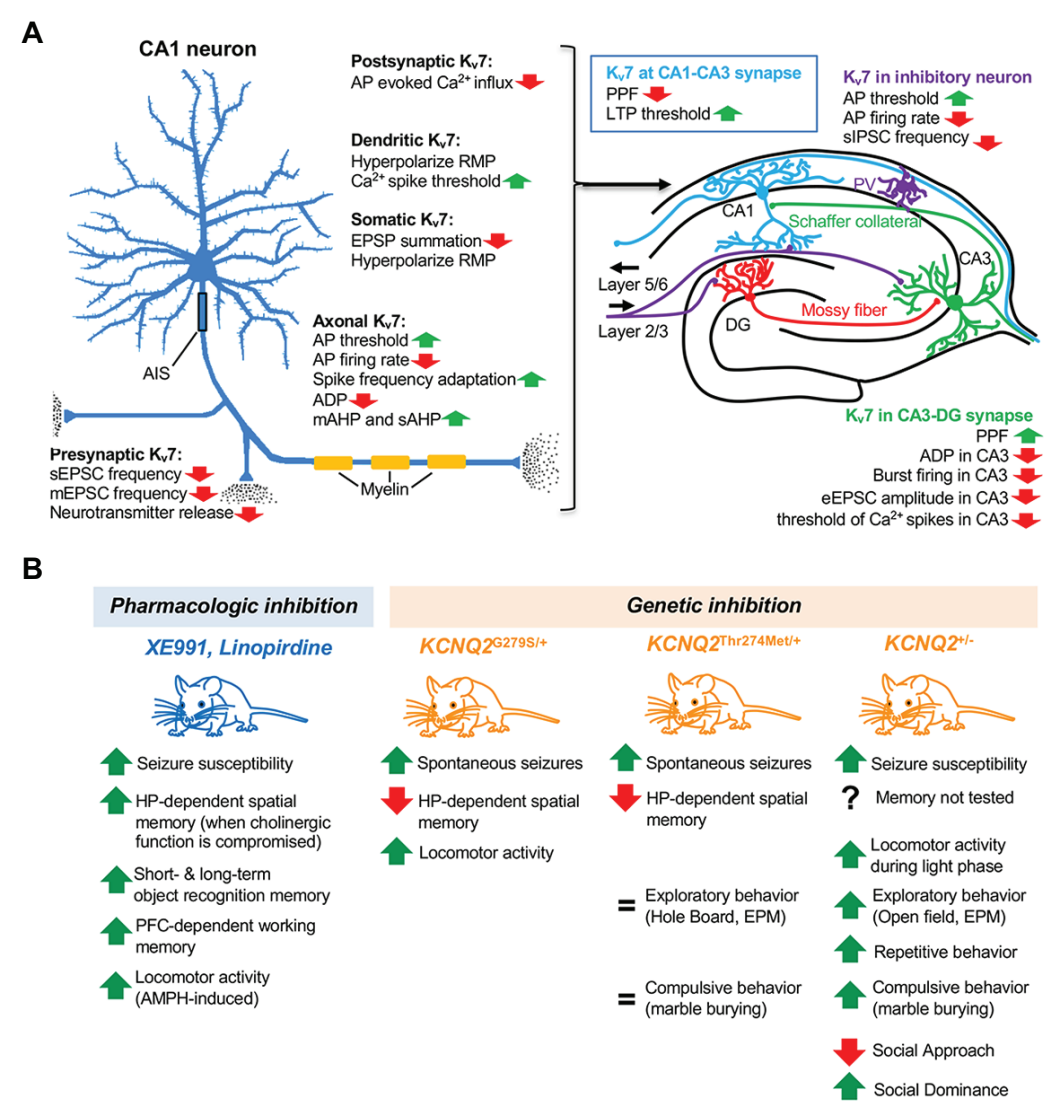
The role of K_v_7 channels in hippocampal neurons, memory, and behavior. **(A)** Function of K_v_7 channels in excitatory pyramidal neuron and GABAergic inhibitory neuron in the hippocampus. AP, action potential; RMP, resting membrane potential; EPSP, excitatory postsynaptic potential; ADP, afterdepolarization; mAHP, medium afterhyperpolarization; sAHP, slow afterhyperpolarization; sEPSC, spontaneous excitatory postsynaptic current; mEPSC, miniature excitatory postsynaptic current; PPF, paired-pulse facilitation; LTP, long-term potential; sIPSC, spontaneous inhibitory postsynaptic current; PV, parvalbumin; and eEPSC, evoked excitatory postsynaptic current. **(B)** Effects of pharmacological or genetic inhibition of K_v_7 channels on memory and behavior. HP, hippocampus; PFC, pre-frontal cortex; AMPH, amphetamine; and EPM, elevated plus maze.

Brown and Adams have first reported in 1980 that inhibition of *I*_M_ upon stimulation of muscarinic acetylcholine receptor results in repetitive firing of APs in bullfrog sympathetic ganglion neurons (Brown and Adams, 1980). In the hippocampus, strong expression of K_v_7.2, K_v_7.3, and K_v_7.5 is detected in pyramidal neurons (Schroeder et al., 2000; Cooper et al., 2001; Devaux et al., 2004). K_v_7 antagonists XE991 and linopirdine depolarize resting membrane potential (RMP) and reduce AP threshold of hippocampal CA1 pyramidal neurons, resulting in spontaneous AP firing (Aiken et al., 1995; Shah et al., 2008; Figure 1A). K_v_7 antagonists also increase intrinsic excitability (Yue and Yaari, 2004, 2006; Shah et al., 2008), contribute to medium and slow afterhyperpolarization (AHP) currents (Gu et al., 2005), reduce spike frequency adaptation (Aiken et al., 1995), and ultimately lead to an increased AP firing rate (Lezmy et al., 2020; Figure 1A). Consistent with pharmacologic inhibition, suppression of K_v_7 current by overexpressing K_v_7.2 containing dominant-negative pore mutation G279S enhances intrinsic excitability and reduces spike frequency adaptation and mAHP in CA1 neurons (Peters et al., 2005). Similarly, conditional homozygous deletion of *KCNQ2* increases CA1 excitability due to longer-lasting spike afterdepolarization (ADP) and reduced medium AHP (Soh et al., 2014; Figure 1A). Thus, K_v_7 channels serve as critical “brakes” on neuronal excitability (Soldovieri et al., 2011).

The inhibitory effects of K_v_7 currents on neuronal excitability are largely attributed to axonal K_v_7 channels. K_v_7.2/K_v_7.3 channels are preferentially enriched at the axonal plasma membrane compared to the somatodendritic plasma membrane in hippocampal neurons (Chung et al., 2006) with the highest concentration at the axonal initial segments (AIS; Chung et al., 2006; Pan et al., 2006) where AP initiates (Clark et al., 2009). CaM binding to K_v_7.2 is critical for targeting K_v_7.2/K_v_7.3 channels to the axonal surface (Cavaretta et al., 2014), whereas disruption of this binding decreases *I*_M_ and increases hippocampal neuronal excitability (Shahidullah et al., 2005). Furthermore, disrupting the enrichment of K_v_7 channels at the AIS by blocking their interaction with ankyrin-G results in spontaneous firing of CA1 neurons by depolarizing RMP and reducing AP threshold (Shah et al., 2008).

In contrast to the well-documented function of axonal K_v_7 channels discussed above, the existence and role of dendritic K_v_7 channels are still in debate. Non-inactivating K_v_7 current sensitive to muscarinic agonist is detected in the distal apical dendrites of CA1 neurons (Chen and Johnston, 2004). Dendritic K_v_7 current can increase the threshold for initiating calcium (Ca^2+^) spikes and induce spike bursts only in hyperexcitable conditions that promote Ca^2+^ electrogenesis in these dendrites (Yue and Yaari, 2006). However, XE991 and linopirdine do not affect input resistance of CA1 dendrites (Shah et al., 2008), and focal inhibition of dendritic *I*_M_ has no effect on the excitatory postsynaptic potential (EPSP) summation and excitability of CA1 neuron (Hu et al., 2007), indicating very low level of dendritic K_v_7 current.

There is accumulating evidence for activity-dependent modulation of K_v_7 channels and their contribution to persistent changes in intrinsic excitability termed “intrinsic plasticity.” In the pilocarpine model of temporal lobe epilepsy, reduced K_v_7 function and expression may contribute to muscarinic-dependent ictogenesis (Maslarova et al., 2013). However, acute induction of seizures increases *KCNQ2* and *KCNQ3* transcripts in the hippocampi as a homeostatic response to suppress neuronal hyperexcitability, and this regulation requires activation of L-type voltage-gated Ca^2+^ channels (Zhang and Shapiro, 2012). Enhancing neuronal activity by K_v_7 inhibition with XE991 also results in homeostatic suppression of firing rate over 48 h (Lezmy et al., 2020). In contrast, prolonged blockade of neuronal activity or N-methyl-D-aspartate (NMDA) receptors increases firing rate and reduces in *KCNQ3* transcript and K_v_7 current in hippocampal neurons (Lee and Chung, 2014; Lee et al., 2015). In the avian cochlear neurons, depriving afferent inputs induces a switch from fast activating K_v_1 to slow activating K_v_7.2 channels at the AIS, resulting in enhanced excitability (Kuba et al., 2015). This activity-dependent regulation of K_v_7 transcript and distribution offers a powerful means to control intrinsic excitability.

## Role of K_v_7 Channels in Synaptic Transmission and Plasticity in the Hippocampus

Since the discovery of long-term potentiation (LTP) in the dentate gyrus of the hippocampus (Bliss and Lomo, 1973), persistent modification in synaptic strength termed “synaptic plasticity” has attracted significant attention as the cellular correlate of learning and memory (Nicoll, 2017). LTP at excitatory synapses can exert destabilizing influence on neural circuits by generating unconstrained synaptic strengthening (Turrigiano, 2012). Homeostatic plasticity counteracts such destabilizing condition by allowing neurons to adjust their synaptic strength (Turrigiano, 2012). While activity-dependent modulation of glutamate release and glutamate receptors serves as key mechanisms for LTP expression (Turrigiano, 2012; Humeau and Choquet, 2019), K_v_7 channels and upstream muscarinic acetylcholine receptors have emerged as important regulators of excitatory synaptic transmission and plasticity.

Synaptic functions of K_v_7 channels have been extensively studied at the excitatory synapses formed by hippocampal CA1 and CA3 pyramidal neurons (Figure 1A). These neurons show strong expression of K_v_7.2 and K_v_7.3 (Cooper et al., 2001; Pan et al., 2006). Conditional deletion of *KCNQ2* and *KCNQ3* increases the frequency of spontaneous excitatory postsynaptic currents (EPSC) in CA1 neurons (Soh et al., 2018), suggesting enhanced presynaptic release at CA1–CA3 synapses. Consistent with this notion, application of K_v_7 antagonist XE991 increases whereas K_v_7 agonist Flupirtine decreases miniature EPSC frequency in CA1 neurons (Sun and Kapur, 2012). Furthermore, K_v_7 inhibition with linopirdine and XE991 treatment also increases neurotransmitter release (Nickolson et al., 1990; Martire et al., 2004; Peretz et al., 2007). While K_v_7 current restrains AP-evoked Ca^2+^ influx into the presynaptic terminal and decreases the paired pulse ratio of evoked EPSCs at the mossy fiber–CA3 synapses (Martinello et al., 2019), paired pulse facilitation of EPSP is higher at CA1–CA3 synapses in XE991-treated mice (Fontan-Lozano et al., 2011), suggesting differential roles of K_v_7 channels in short-term plasticity at two different synapses.

K_v_7.2 and K_v_7.3 are expressed in GABAergic neurons including parvalbumin (PV)- and somatostatin (SST)-positive interneurons in the hippocampus (Cooper et al., 2001; Lawrence et al., 2006). Application of XE991 abolishes *I*_M_, depolarizes RMP, and increases AP firing in SST+ interneurons (Lawrence et al., 2006) and enhances intrinsic excitability of PV+ interneurons (Soh et al., 2018; Figure 1A). Furthermore, conditional deletion of *KCNQ2* and *KCNQ3* from PV+ interneurons increases their firing and spontaneous inhibitory postsynaptic current (sIPSC) frequency of CA1 neurons in the hippocampus (Soh et al., 2018).

These studies highlight the presynaptic influence of K_v_7 channels at glutamatergic and GABAergic synapses. Given that increased firing rate and burst firing can enhance neurotransmitter release probabilities (Hansen et al., 2008), K_v_7 inhibition may increase neurotransmitter release as a consequence of increased axonal excitability (Devaux et al., 2004; Shah et al., 2008; Klinger et al., 2011). Indeed, when CA3 neurons are depolarized upon elevating extracellular K^+^ concentration, XE991 enhances EPSP amplitude in CA1 neurons as a consequence of increasing spike ADP and burst firing of CA3 neurons (Vervaeke et al., 2006). Alternatively, K_v_7 channels at the presynaptic terminals (Cooper et al., 2001; Martire et al., 2004; Regev et al., 2009) may directly counteract the depolarization of the presynaptic membrane necessary for synaptic vesicle fusion and neurotransmitter release.

The postsynaptic role of K_v_7 channels is unclear. A recent electron microscopy study shows that K_v_7.2, K_v_7.3, and K_v_7.5 colocalize with muscarinic acetylcholine receptors at dendritic spines in layer III pyramidal neurons of the primate prefrontal cortex (Galvin et al., 2020), although the specificity of the immunolabeling needs to be further validated. In the CA1–CA3 synapses, the mEPSC amplitude is unaltered by agonist nor antagonists of K_v_7 channels (Sun and Kapur, 2012), suggesting their negligible role in regulating postsynaptic glutamate receptor function at this synapse (Figure 1A).

Nonetheless, accumulating evidence suggests that K_v_7 channels regulate hippocampal synaptic plasticity. At CA1–CA3 synapses, XE991 induces LTP by subthreshold theta-burst stimulation (Petrovic et al., 2012). Systemic administration of XE991 also decreases the threshold for LTP induction in the hippocampal CA1 area *in vivo* without affecting the field EPSP amplitude (Song et al., 2009; Fontan-Lozano et al., 2011). Lastly, homeostatic increase in excitatory synaptic transmission in CA1 neurons has been observed upon conditional deletion of *KCNQ2* and *KCNQ3* from GABAergic interneurons (Soh et al., 2018), suggesting the contribution of K_v_7 channels in synaptic scaling.

## Role of K_v_7 Channels in Hippocampus-Dependent Learning and Memory

Hippocampal LTP occurs during hippocampus-dependent learning and memory (Bliss et al., 2018) and its reduction is linked to memory loss in mouse models of Alzheimer’s disease (Mango et al., 2019). Facilitation of LTP induction by XE991 (Song et al., 2009; Fontan-Lozano et al., 2011; Petrovic et al., 2012) suggests that pharmacologic K_v_7 inhibition may enhance learning and memory. Indeed, linopirdine enhances the performance of rats in a hippocampus-dependent active avoidance test (Cook et al., 1990). XE991 improves memory in object recognition task in wild-type mice and mouse models of dementia induced by cholinergic depletion and neurodegeneration (Fontan-Lozano et al., 2011; Ballinger et al., 2016; Dennis et al., 2016) despite its ability to induce seizures at a higher dose (Fontan-Lozano et al., 2011; Figure 1B). In contrast, K_v_7 agonists have yielded mixed results on affecting memory in rodents (Li et al., 2014; Frankel et al., 2016).

The cognition-enhancing effect of linopirdine is correlated with the increased release of acetylcholine in the hippocampus (Nickolson et al., 1990; Fontana et al., 1994), and stimulation of muscarinic acetylcholine receptor inhibits *I*_M_ in hippocampal neurons (Shah et al., 2002). Consistently, muscarinic agonist improves whereas anticholinergic agent scopolamine impairs performance in hippocampus-dependent memory tasks (Fontana et al., 1994; Fontan-Lozano et al., 2011). Muscarinic acetylcholine receptors in the prefrontal cortex also modulate working memory in primates *via* K_v_7 channels (Galvin et al., 2020). Since cholinergic depletion and dysfunction in the hippocampus and prefrontal cortex are implicated in age-related cognitive decline and Alzheimer’s disease (Ballinger et al., 2016; Haam and Yakel, 2017), these studies support the therapeutic potential for K_v_7 antagonists as cognitive enhancers.

Surprisingly, genetic inhibition or reduction of K_v_7 currents induces an opposite effect on memory (Figure 1B). Deficits in hippocampal-dependent spatial memory and spontaneous seizures are observed in mice with conditional transgenic expression of dominant-negative mutant K_v_7.2-G279S (Peters et al., 2005) and heterozygous knock-in mice for K_v_7.2 containing epileptic encephalopathy loss-of-function variant T274M (Milh et al., 2020). Considering that K_v_7 channels are critical for development and inhibition of neonatal brain (Peters et al., 2005; Soh et al., 2014), the memory impairment in these genetic models could be attributed to abnormal hippocampal morphology and/or hyperexcitability (Peters et al., 2005; Milh et al., 2020).

K_v_7 channels also regulate multiple behaviors (Figure 1B). Behavioral phenotyping of the global or conditional homozygous *KCNQ2* knock-out mice has not been possible due to their early postnatal lethality or premature death, respectively (Watanabe et al., 2000; Soh et al., 2014). However, heterozygous *KCNQ2* knock-out mice are viable and display increased locomotor activity and exploratory behavior (Kim et al., 2020), consistent with behavioral hyperactivity induced by transgenic suppression of K_v_7 currents (Peters et al., 2005) and amphetamine and XE991 (Sotty et al., 2009). These mice also show decreased sociability and increased repetitive and compulsive behavior (Kim et al., 2020), reminiscent of autism seen in some EE patients with dominant *KCNQ2* mutations (Weckhuysen et al., 2012, 2013; Milh et al., 2013). However, the precise circuitries responsible for these abnormal behaviors remain unknown.

## Future Perspectives

The studies discussed in this review support the emerging concept that K_v_7 channels contribute to neural plasticity, memory, and behavior. However, there is a significant knowledge gap in our understanding of the underlying molecular and cellular mechanisms. Future studies should continue to investigate structure-function and subcellular targeting of K_v_7 channels, which will provide mechanistic insights for developing specific modulators of their function and trafficking. Generation of mouse models in which deletion of a K_v_7 subunit from specific neurons and subcellular localization with temporal control will be critical to delineate cell- and circuit-specific function of K_v_7 channels in neural plasticity, cognition, and behavior.

## Author Contributions

BCB and HC contributed to the conception and design of the manuscript. BCB, JZ, and HC also drafted and revised the manuscript. All authors contributed to the article and approved the submitted version.

### Conflict of Interest

The authors declare that the research was conducted in the absence of any commercial or financial relationships that could be construed as a potential conflict of interest.
